# Phytochemicals in Food and Health

**DOI:** 10.3390/foods10040901

**Published:** 2021-04-20

**Authors:** Dilip K. Rai

**Affiliations:** Department of Food BioSciences, Teagasc Food Research Centre Ashtown, D14K N3K Dublin, Ireland; dilip.rai@teagasc.ie

Consumption of plant-based diets, rich in phytochemicals, has been associated with reduced risk of degenerative diseases, improved overall health and well-being. However, assigning and identifying the food components, i.e., specific phytochemical and/or classes of phytochemicals, responsible for their biological activities are not adequately addressed. In addition, the impacts of thermal and emerging non-thermal food processing methods on the fate of phytochemicals are scarce. In this special issue, phytochemicals responsible for various biological activities have been investigated with advanced analytical techniques including liquid chromatography tandem mass spectrometry (LC–MS/MS), nuclear magnetic resonance (NMR) spectroscopy and molecular-docking experiments [1,2,3,4,5]. The impact of domestic thermal processing and non-thermal (high-pressure processing) on the phytochemicals has also been covered. Here, the roasting of broomcorn millet (*Panicum miliaceum* L.) significantly enhanced the antioxidant polyphenols, in particular ferulic acid, as opposed to steaming, puffing and extrusion [6]. High-pressure processing at 600 MPa for 3 min of potatoes significantly reduced the polyphenol oxidase activity and the polyphenol responsible for potato browning, i.e., chlorogenic acid, without impacting antioxidant capacity as well as the cytotoxic glycoalkaloids (α-solanine and α-chaconine) contents [5]. Caffeic acid derivatives, in particular salvianic acid A, from the leaves of *Actinidia arguta* (Hardy Kiwi) have been linked for anti-inflammatory properties [2]. Anti-inflammatory potency of commercial phytosterols, where β-sitosterols were more effective than stigmasterols and campesterol, has been established [7]. Triterpenoids in *Minthosa diffusa* play key role in the inhibition of cholinesterases—the enzymes associated with Alzheimer’s and Parkinson’s diseases, and may also contribute against diabetes [4]. On the other hand, individual or a category of polyphenols from different berries could not be clearly linked to various biological activities, suggesting the interplay of different polyphenols in promoting health, and therefore requires further studies [3,8].
